# Supplementary material for: Evaluation of Rapid Lateral-Flow Tests Directed against the SARS-CoV-2 Nucleoprotein Using Viral Suspensions Belonging to Different Lineages of SARS-CoV-2
Source: Viruses. 2022 Nov 25;14(12):2628. doi: 10.3390/v14122628 (PMC9787475; doi:10.3390/v14122628)
Supplement: Supplementary file 1 [file viruses-14-02628-s001.zip › viruses-2002487-supplementary.pdf]

Supplementary Table S1. Series five-fold dilutions of different strains belonging to 7 strains of SARS-CoV-2 tested by 4 rapid lateral-flow tests (see main text) and a true quantitative RT-PCR test. Regarding the latter test, the last right columns illustrate the correspondence between the CT value and the actual viral load.

Strain lineage	Biospeedia	BD	Abbott	AAZ	Nucleocapsid gene amplification (C_T value)	Viral load (copies/200µl of viral culture)
19A	Positive	Positive	Positive	Positive	20.69	6.45
	Positive	Positive	Positive	Positive	23.32	5.68
	Positive	Positive	Positive	Positive	24.65	5.28
	Weak positive	Negative	Weak positive	Weak positive	28.64	4.10
	Negative	Negative	Negative	Very weak positive	30.80	3.46
	Negative	Negative	Negative	Negative	31.93	3.13
20A	Positive	Positive	Positive	Positive	21.08	6.34
	Positive	Positive	Positive	Positive	23.28	5.69
	Positive	Negative	Positive	Positive	25.32	5.08
	Weak positive	Negative	Weak positive	Negative	27.86	4.33
	Negative	Negative	Negative	Negative	30.12	3.67
Alpha	Positive	Positive	Positive	Positive	21.07	6.34
	Positive	Positive	Negative	Positive	23.10	5.74
	Positive	Negative	Positive	Negative	24.47	5.34
	Weak positive	Negative	Positive	Negative	26.99	4.59
	Negative	Negative	Negative	Negative	30.16	3.65
Beta	Positive	Positive	Positive	Positive	23.23	5.70
	Positive	Positive	Positive	Positive	25.56	5.01
	Weak positive	Negative	Weak positive	Positive	29.07	3.97
	Negative	Negative	Negative	Negative	31.23	3.34
Gamma	Positive	Positive	Positive	Positive	21.93	6.09
	Positive	Positive	Positive	Positive	24.45	5.34
	Weak positive	Negative	Positive	Weak positive	26.96	4.60
	Very weak positive	Negative	Negative	Negative	28.97	4.00
	Negative	Negative	Negative	Negative	31.51	3.25
Delta	Positive	Positive	Positive	Positive	19.84	6.70
	Positive	Positive	Positive	Positive	22.32	5.97
	Positive	Positive	Positive	Negative	24.95	5.19
	Weak positive	Negative	Weak positive	Negative	27.03	4.58
	Negative	Negative	Negative	Negative	29.17	3.95
Omicron	Positive	Positive	Positive	Positive	22.03	6.06
	Positive	Positive	Positive	Positive	23.98	5.48
	Weak positive	Negative	Weak positive	Weak positive	26.34	4.78
	Negative	Negative	Negative	Negative	28.85	4.04
